# A Validation Study of a Deep Learning-Based Doping Drug Text Recognition System to Ensure Safe Drug Use among Athletes

**DOI:** 10.3390/healthcare11121769

**Published:** 2023-06-15

**Authors:** Sang-Yong Lee, Jae-Hyeon Park, Jiwun Yoon, Ji-Yong Lee

**Affiliations:** Center for Sports and Performance Analysis, Korea National Sport University, Seoul 05541, Republic of Korea; 20220018@m365.knsu.ac.kr (S.-Y.L.); jhpark@knsu.ac.kr (J.-H.P.); jwyoon@knsu.ac.kr (J.Y.)

**Keywords:** banned substances, doping, Korea, optical character recognition

## Abstract

This study aimed to develop an English version of a doping drug-recognition system using deep learning-based optical character recognition (OCR) technology. A database of 336 banned substances was built based on the World Anti-Doping Agency’s International Standard Prohibited List and the Korean Pharmaceutical Information Center’s Drug Substance Information. For accuracy and validity analysis, 886 drug substance images, including 152 images of prescriptions and drug substance labels collected using data augmentation, were used. The developed hybrid system, based on the Tesseract OCR model, can be accessed by both a smartphone and website. A total of 5379 words were extracted, and the system showed character recognition errors regarding 91 words, showing high accuracy (98.3%). The system correctly classified all 624 images for acceptable substances, 218 images for banned substances, and incorrectly recognized 44 of the banned substances as acceptable. The validity analysis showed a high level of accuracy (0.95), sensitivity (1.00), and specificity (0.93), suggesting system validity. The system has the potential of allowing athletes who lack knowledge about doping to quickly and accurately check whether they are taking banned substances. It may also serve as an efficient option to support the development of a fair and healthy sports culture.

## 1. Introduction

During the Beijing 2022 Winter Olympics, female figure skater Kamila Valieva under the Russian Olympic Committee (ROC) tested positive for a banned substance. The Court of Arbitration for Sport (CAS) ruled, under the World Anti-Doping Code by the World Anti-Doping Agency (WADA), that the figure skater lacked the capacity to control her drug use at her young age—she was 16 years old at the time. Following the ruling, Valieva was permitted to compete in the Beijing 2022 Winter Olympics, sparking one of the biggest controversies that occurred during the Olympics [1].

As is exemplified in Valieva’s case, doping, that is, the use of banned drugs or other substances to enhance performance, is recognized as a serious problem in sports worldwide, irrespective of country, age, gender, and other factors [2]. In Korea, former Korean swimmer Park Tae-hwan, who won four Olympic medals, two world titles, and more than twelve Asian Games medals, tested positive for the banned substance testosterone while taking medication to treat an injury; this led the Fédération Internationale de Natation to notify him of a violation of doping regulations, and he was eventually suspended for 18 months [3]. As another example, Lance Armstrong, who was once a legendary cyclist and a seven-time winner of the Tour de France (one of the most authoritative cycling races), was discovered to have used banned substances by the United States Anti-Doping Agency in August 2012. This led him to be stripped of all his athletic accomplishments since 1998 [4]. There have also been doping cases across other sports and competitions, including Major League Baseball in the 1990s, often known as the steroid era, when some of the greatest baseball players of all time, such as Barry Bonds and Mark McGwire, were found to have used banned substances [5,6,7].

In sports, doping is cheating and must be banned to protect athletes because taking these banned substances leads to improved performance and can cause negative side effects [8]. Considering these descriptions and the risks involved, why do athletes continue to dope? The scientific evidence shows that there is a constant temptation to dope for better performance among athletes competing in international competitions. Previous studies on why athletes take risks and use doping or banned substances found that some of the fundamental reasons are to enhance their performance for specific purposes, such as controlling body weight to move to a lower weight class or achieving an overwhelming victory over an opponent [9]. The use of banned substances, such as steroids and testosterone, has a significant effect on performance [10,11]. Furthermore, athletes feel anxious about incurring an unprepared retirement because of external factors, including injury and declining performance, with the latter often coming earlier for athletes than people in other professions [12]. Baron et al. [13] also found that athletes may rely on substances to cope with various stress factors, including injury, physical pain, and retirement from their athletic careers. Regardless of the reason, as mentioned, doping to enhance performance must be prohibited as it constitutes a violation of the principle of fair play and poses risks to athletes’ health, including death.

In 1999, the WADA was created to promote, coordinate, and monitor efforts to prevent doping, which can be potentially life-threatening to athletes, and this subsequently led athletes and sports all over the world to become subject to tighter control and regulations [14]. The WADA created the International Standard Prohibited List, which is updated every September with the drugs or methods considered to harm an athlete’s performance or health, and the updated list takes effect from 1 January of the following year [15]. The WADA also takes the lead in protecting athletes from the intake of banned substances by competitors by annually conducting regular doping tests on athletes across different countries and providing data on cases where doping is discovered [16]. Consequently, doping tests are becoming increasingly frequent and have been strengthened to accurately detect even trace amounts of banned substances [17]. Nonetheless, despite efforts by the WADA and national anti-doping organizations, positive doping test results still persist worldwide [18]. Research also shows that methods to evade doping tests have continued to develop and generally keep up with advances in science and technology for such tests, allowing athletes to get away with the intake of banned substances even with frequent testing measures [19].

At this point, a question arises: are all doping violations, which are discovered consistently, only organized and intentional? Past studies show that some athletes described a lack of awareness and knowledge about doping tests and the WADA’s regulations [20,21]. In addition, substances in prescription drugs provided by hospitals and over-the-counter drugs purchased directly from pharmacies are often written in a manner difficult for athletes to understand, and they may take the drugs without knowing about the banned substances or applicable regulations. To address these issues, it is crucial to provide athletes with accurate information through anti-doping training from a young age and to support the ban on the use of performance-enhancing drugs [22,23,24]. Furthermore, athletes do not know a lot about regulations on banned substances, which continues to cause problems such as redundant and excessive prescriptions. To prevent these problems, athletes, coaches, agents, and pharmacists (who can prescribe drugs) need to receive specialized training on doping [25,26].

Scholars have also recently proposed the use of optical character recognition (OCR) technology to solve these issues, which understands and scans handwritten or printed documents and images to convert and save to Korean, English, and numbers into texts [27]. OCR is a type of deep learning technique [28], in which computers learn about how people think [29]. Although it started out by comparing typed characters with standard patterned characters and recognizing similar ones, OCR has evolved to a point where the software recognizes the spacing of characters in a photographed document and automatically organizes them into words [30]. These technological advances allowed for OCR to be currently used across various contexts, including license plates, bills, and receipts [31,32]. The application of this technique has also been examined in the field of medical text recognition, including handwritten prescription recognition, medical report analysis, and drug identification, to make information about substances in a drug more easily accessible to the public [33,34,35,36].

Previous studies in the field of OCR-based prescription recognition developed systems wherein users can upload photos and check for drug substances or redundant prescriptions. As previously mentioned, it may be difficult for some athletes to identify banned substances when reading the text on a prescription. Thus, building on the aforementioned research, it would be optimal for a doping drug-recognition system to be able to recognize substances in prescriptions, and then compare and analyze these recognized substances with the list of banned substances provided by the WADA. Such a system could potentially help athletes easily check whether the drugs they are prescribed or about to take contain any substance that potentially violates anti-doping regulations. Park et al. [37] developed a doping drug-recognition system to identify banned substances using the images of prescriptions and drug substances in the Korean language. They showed that it had a high accuracy (about 92%) in classifying banned drugs and acceptable drugs. Since the accuracy was calculated with a relatively small number of images, the authors suggested that a larger number of drug substance images should be collected to validate and confirm the practical usability of the doping drug-recognition system. Regarding the limitations of the system they developed, Park et al. [37] mentioned that it is difficult for the system to recognize characters in English prescriptions and drug substance images as its database is built based on the Korean language. Therefore, I decided to conduct a follow-up study to modify and supplement the doping drug-recognition system developed by Park et al. [37]. Specifically, the current study aimed to solve the issue of limited data collection by utilizing data augmentation, and develop a doping drug-recognition system by building an English-based database.

The purpose of this study is to develop an English version of the doping drug-recognition system with deep learning-based OCR technology. The developed system, if used in practical settings in the near future, may allow athletes who lack knowledge about doping to quickly and accurately check whether they are about to take or are taking banned substances. In addition, the system is expected to serve as an efficient option to create a fair and healthy sports culture.

## 2. Materials and Methods

### 2.1. Data

#### 2.1.1. Test Data

**Figure 1:**
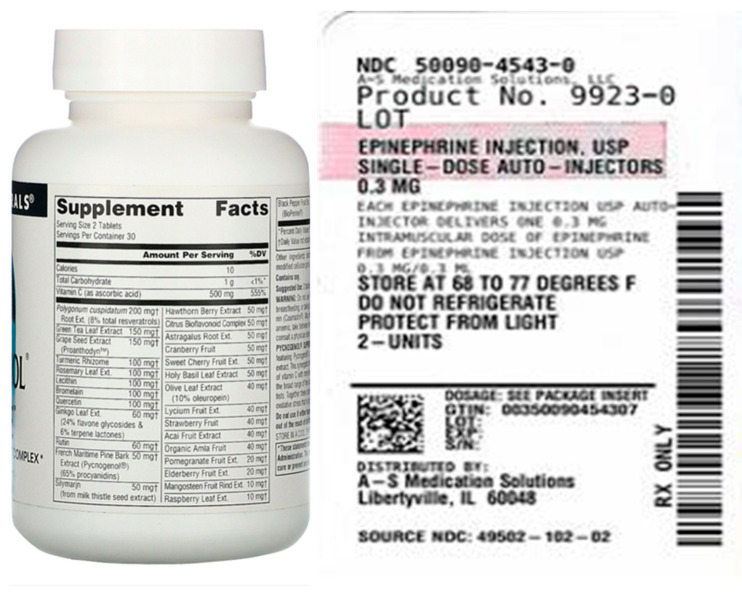
Exclusion criteria for study data.

Data on 200 English prescriptions and drug substance labels were collected using a search engine (Google), the WADA’s International Standard Prohibited List [15], and the Korea Pharmaceutical Information Center’s Drug Substance Information (www.health.kr (accessed on 20 March 2023); Table 1). In total, 152 images were selected as the primary study data (Figure 1), excluding 18 images considered unanalyzable due to image quality, angles, and substances labeled on round containers, as well as 30 images used in a preliminary test.

#### 2.1.2. Data Augmentation

Image augmentation is one of the most widely used data-augmentation techniques in machine learning and computer vision. The most common method of image augmentation is to transform images by rotating, moving, scaling, or flipping them [38]. It is mainly used to improve the performance of a model in image classification, object detection, and segmentation. In particular, when the amount of training data is small, the technique can be useful for improving the accuracy of a model by improving a model’s performance and preventing overfitting [39]. Hence, the collected primary data of 152 images was augmented with five images (data) each using the image augmentation technique.

During this process, the author set the minimum default values for rotation, scaling, magnification, and color to make characters recognizable for the purpose of the study. After excluding 26 images in which substances were deemed difficult to analyze due to image quality, angle, or other reasons, a total of 886 images, including the above 152 images, were selected as the final study dataset.

#### 2.1.3. Database

The system developed in this study requires a database that can identify banned drugs and confirm whether banned substances are included in drugs when recognizing drug substances using OCR technology. This study analyzed the 152-test data based on the WADA’s International Standard Prohibited List [15], and constructed a database containing 336 banned substances.

### 2.2. Optical Character Recognition-Based Doping Drug-Recognition System

#### 2.2.1. Composition and Mechanism

**Figure 2:**
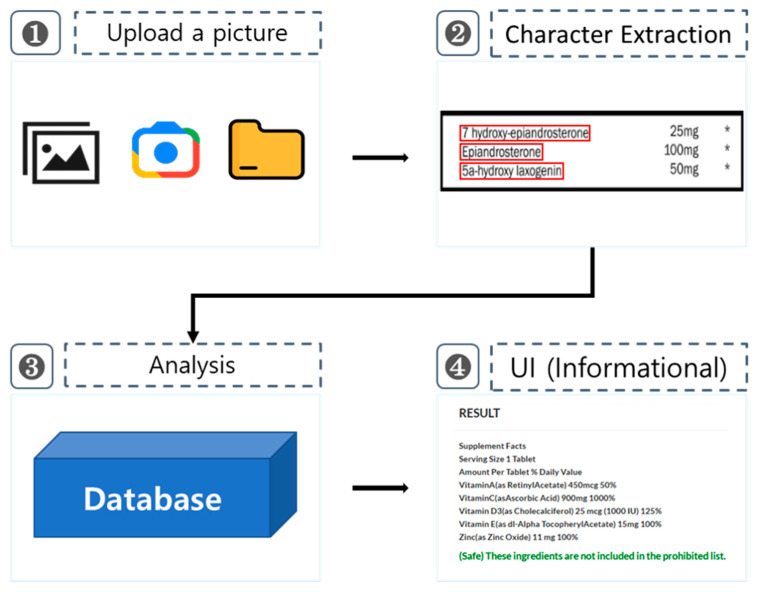
Overview of the optical character recognition-based doping drug-recognition system.

The OCR-based doping drug-recognition system developed in this study consists of four steps (Figure 2). In Step 1, a photo is taken with a smartphone camera or uploaded to recognize characters in the image containing drug substances. In Step 2, characters are automatically extracted by OCR from the photographed or uploaded photo. In Step 3, the system checks whether the extracted drug substances contain banned substances or are safe to take by comparing them with the list of banned drugs in the database. In Step 4, the analyzed results are provided to the user in the user interface (UI). The UI was built as a hybrid system accessible by both a smartphone and website.

**Figure 3:**
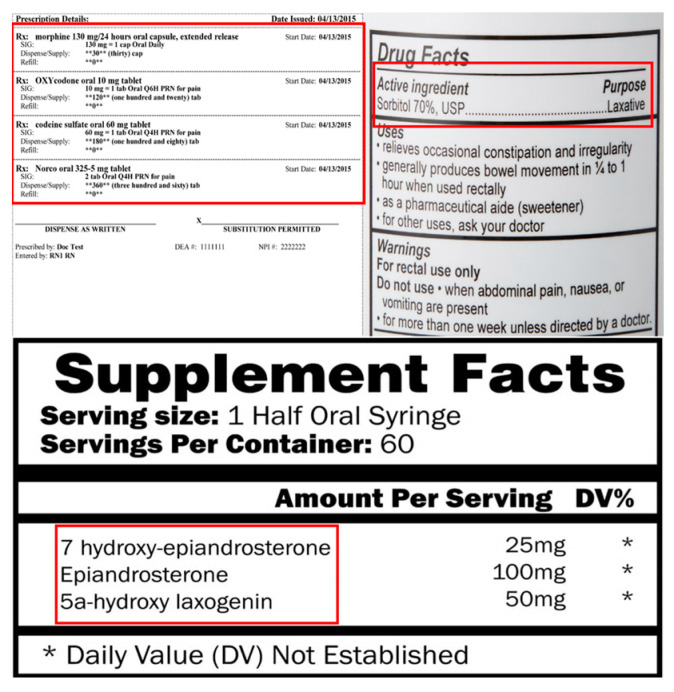
Sections of prescriptions and drug labels to be recognized by the system.

To improve the accuracy of the OCR-based doping drug-recognition system developed in this study, the system was built in a way that automatically saves the analyzed photos and results, so that the researcher can determine any error. Specifically, the system used a section of a label containing the active ingredient of a drug, such as drug facts or prescription drug name (Figure 3), as information about substances in drugs that athletes may want to check. In addition, images of a section of a label containing the active ingredient were entered, and Tesseract OCR was used to extract text from the designated section.

#### 2.2.2. Tesseract OCR

The Tesseract OCR engine is an open-source software developed by HP between 1985 and 1994. In 2005, it was released as an open-source project; following this, it has been supported by Google since 2006, alongside continuous performance improvements [40]. The Tesseract OCR offers efficient performance and scalability, making it useful in various fields such as document processing, data mining, automatic license plate recognition, automated data entry, analysis of advertisements and promotional materials, and translation [41]. Furthermore, it supports character recognition in various image formats, including JPEG, PNG, and TIFF. However, the recognition results may vary depending on factors such as the quality and resolution of the original image, noise, font types, and background [42]. To address these issues, this study constructed a database by utilizing preprocessing techniques such as defining the recognition range of the desired images, improving resolution, and removing noise. Specifically, in the doping substance recognition system, which is based on OCR, athletes rely on the provided information area or the prescription drug name, as presented in Figure 3, to obtain the valid components of the medication. The Tesseract OCR was used while inputting images containing the relevant area and extracting the text from the designated region.

The Tesseract OCR engine does not require its own page layout analysis because HP independently developed proprietary page layout analysis technology, which was utilized in their products but not released as open source. Consequently, Tesseract assumes that its input is a binary image with optional polygonal text regions defined. The processing follows a traditional step-by-step pipeline [41].

The initial step involves connected component analysis, where the outlines of the components are stored. This design decision was computationally expensive at the time but had a significant advantage. By examining the nesting of outlines and the number of child and grandchild outlines, Tesseract can easily detect inverse text. The outlines are gathered together into blobs based solely on their nesting relationships.

In summary, the architecture of the Tesseract OCR engine relies on externally developed page layout analysis technology, enabling it to process binary images with optional text regions. It follows a step-by-step pipeline, with connected component analysis being a unique and computationally intensive stage. This approach allows for the detection and recognition of inverse text as easily as black-on-white text. Research findings on the Tesseract OCR engine can be found in the work of Smith, R. [41].

#### 2.2.3. Text Recognition by Tesseract OCR

The Tesseract OCR model developed by Google was used for system development. The accuracy of character recognition was checked in a preliminary test to determine whether it was an acceptable model for the study purposes (Table 2).

A preliminary test extracting 323 words from a total of 30 images of drug substances showed a character recognition accuracy of approximately 96.3%, with 311 correct recognitions and 12 errors. Therefore, Google’s Tesseract OCR model was considered acceptable for system development in this study.

The following describes the detailed process performed by the OCR technology. First, prescription and drug substance label images are entered, binarized, and preprocessed before recognition. Binarization can maximize OCR performance by removing curvature or noise. Second, segmentation is performed to create a system that can handle each character individually. Third, the segmented characters are classified and recognized.

### 2.3. Data Processing and Analysis Method

To validate the doping drug-recognition system, the author calculated the classification accuracy, sensitivity, and specificity using a binary classification table. Accuracy refers to the frequency at which banned drugs are correctly recognized (true master [TM]) and acceptable drugs are correctly recognized (true non-master [TN]) in all prescription and drug substance images. Sensitivity refers to the percentage of banned substances’ images that are correctly classified as banned (TM). Specificity refers to the percentage of acceptable substances’ images that are correctly classified as acceptable (TN). Table 3 provides examples of the accuracy, sensitivity, and specificity calculations used by the doping drug-recognition system for classification. Only an accuracy of 0.50–1.00 is interpreted as meaningful, and an accuracy of 0.80 or higher is generally required for suggesting a desirable level of validity [43].

## 3. Results

### 3.1. Doping Drug-Recognition System

This study developed a doping drug-recognition system using the Tesseract OCR model provided by Google. This hybrid system makes the analyzed results accessible to the user through both a smartphone and website through the UI. System accessibility is important for athletes not totally familiar with the applicable doping regulations to easily identify the banned substances. Accordingly, the procedure of the use of the doping drug-recognition system was divided into four steps (i.e., log in the system, enter a drug substance image, analyze substances after image upload, and produce a text as an output of the analysis), and user convenience was prioritized so that anyone can easily use the system. Consequently, it was observed that the time required for scanning and processing the image to provide a response to the user ranged from 10 to 15 s. Table 4 presents details of the procedure of use of the developed system.

### 3.2. Character Recognition Accuracy in the Developed System

This study confirmed the character recognition accuracy of the developed doping drug-recognition system using 886 images, including prescriptions and drug substance labels. Table 5 lists the frequency and accuracy of words extracted from the images. In total, 5379 words were extracted from the analysis of the images, and the system had recognition errors regarding 91 words. As such, it demonstrated a character recognition accuracy of 98.3%.

### 3.3. Validation of the Doping Drug-Recognition System

The analysis of the 886 acceptable and banned drug substance images revealed that the system correctly classified all 624 images with acceptable substances as acceptable drugs. In addition, it correctly classified 218 out of the 262 images with banned substances as banned drugs, but incorrectly recognized 44 of them as acceptable drugs. To validate the system developed, a binary classification table was used to calculate the accuracy, sensitivity, and specificity of the classifications (Table 6).

The validation showed an accuracy of 0.95, sensitivity of 1.00, and specificity of 0.93. Given that an accuracy of 0.80 or higher describes a desirable level of validity, these results suggest that the developed doping system effectively classifies both banned and acceptable substances. These findings indicate the validity of the system for classifying banned and acceptable substances.

## 4. Discussion

This study is a follow-up to the research conducted by Park et al. [37] and presents progressive results compared to their study. First, it achieved an improvement of approximately 3% in performance based on the Acc metric, surpassing the results of the previous study. Second, it addressed the data collection issue identified in the previous research by utilizing data-augmentation techniques to augment the amount of data. This allowed for the validation of the system using a larger dataset compared to the previous study. Third, the database, which was initially limited to Korean, was redesigned to be an English database—thereby enabling athletes worldwide to utilize the system. Fourth, while the previous study used the CLOVA OCR model, which is suitable for Korean recognition, this study utilized the Tesseract OCR model, which is suitable for English recognition using deep learning-based OCR technology. Based on our findings and past research, the following points can be discussed.

First, deep learning has been applied across various fields, including computer vision, pattern recognition, robotics, autonomous driving, art, and prediction [44]. To effectively use deep learning technologies, it is important to ensure a large amount of training data and learning speed [45]. These descriptions show that the deep learning-based OCR technology used for developing the doping drug-recognition system has already been highly validated in this field and widely used across many other fields. Nevertheless, this study conducted a preliminary test to see how well it could recognize characters in prescriptions and drug substance labels; the final choice of model was Google’s Tesseract OCR. This procedure was implemented in an effort to increase the reliability of the doping drug-recognition system developed in this study.

Second, the author confirmed the character recognition accuracy of the developed doping drug-recognition system using 886 images, including images of prescriptions and drug substance labels. The system showed recognition errors for 91 words (character recognition accuracy, 98.3%). Thus, the system showed a high level of accuracy in recognizing words for drug substances. As the system in this study was developed for determining whether a drug contained a banned substance, it was considered unnecessary for the system to recognize characters other than those for drug substances. Therefore, the system’s character recognition accuracy was analyzed based on characters extracted as words in the drug substance section of images. Hassan et al. [34] reported that using OCR technology alone may cause problems, such as misinterpretation of drug names due to noise, including bad handwriting and scribbling. These authors further suggested that it is important for machine learning to learn various types of handwriting and drug names to generate and recognize new characters. Accordingly, this study built a complete database for developing the doping drug-recognition system. Nonetheless, various of the problems noted in the aforementioned study should still be addressed in the future to ensure the practical applicability of the system. In addition, Google’s Tesseract OCR, which was used in this study, was not able to recognize some special characters. These special characters are better recognized by CLOVA OCR, which is offered by the Korean search engine Naver. Furthermore, tilted characters in images augmented by data augmentation are sometimes recognized in the wrong sentence order depending on the recognized range. It may be possible to correct the recognition errors found in this study related to the 91 words if these problems, highlighted in the previous sentences, are registered and added to the database or as an alias with initial default values.

Third, the system correctly classified all 624 images for acceptable substances and 218 (out of 262) images for banned substances. However, it incorrectly recognized 44 of the banned substances as acceptable drugs. Regarding the validity of the system, results showed a high level of accuracy (0.95), sensitivity (1.00), and specificity (0.93). These findings suggest the validity of the system for classifying banned and acceptable drugs. In total, 152 prescription and drug substance images were selected as the primary data for this study; however, they were collected from a search engine and mostly consisted of drugs that anyone can buy easily on the market. Considering that prescriptions and drugs containing doping substances are not commonly available in the market, this can be considered a limitation of the data collection process.

To improve the performance of the system, increase the accuracy of the model, and compensate for the insufficient sample size, this study applied data augmentation to the images of acceptable and banned drugs (about half as many images as those for acceptable drugs). Notwithstanding, to ensure that the list of all 336 banned substances in the database is correctly classified, additional efforts should be made in the future to compensate for the shortcomings of the system. For example, researchers can collect images of more banned substances in a future study. In addition, if the system developed in this study is continuously updated with the annually revised list of banned drugs and successfully commercialized, it is expected to serve as an effective option to prevent doping among athletes.

## 5. Conclusions and Suggestions

In developing the English version of the doping drug-recognition system using deep learning-based OCR technology, it is hoped that this study helps athletes who lack knowledge about doping to easily and accurately identify whether they are taking banned substances. It is also expected that the system provided by this study supports the development of a fair and healthy sports culture. In the following, the conclusions of this study are described.

First, this study used the Tesseract OCR model provided by Google to develop the system. This yielded a hybrid system that allows for the analyzed results to be accessible to the user by both a smartphone and website through the UI. Furthermore, the procedure of use for the system was divided into four steps (log into the system, enter a drug substance image, analyze substances after uploading an image, and produce the text as an output of analysis), and user convenience was prioritized to enable for anyone to easily use the system.

Second, the author confirmed the character recognition accuracy of the system using 886 images, including images of prescriptions and drug substance labels, showing a high character recognition accuracy of 98.3% (recognition errors for 91 out of 5379 words).

Third, the system correctly classified all images for acceptable substances and most images for banned substances (44 banned substances were incorrectly recognized as acceptable). The results also suggested the validity of the system for classifying banned and acceptable drugs.

In summary, the study developed a doping drug-recognition system to provide an effective and convenient option to support the efforts against the use of banned drugs, which could undermine a fair and healthy competition culture in the sports community and potentially harm the health of athletes. Further research is needed to address some of the issues with the system and improve it to ensure its practical applicability. Specifically, the validity of the system developed in this study maintains a high level, as mentioned in the research findings. However, regarding minor errors that result in providing incorrect information to athletes, they can have a detrimental impact on their careers. Such issues can be addressed through system modifications and enhancements in future research. The 44 errors that occurred in the study’s results were analyzed and categorized into two types based on issues that happened during the segmentation process—15 errors were caused by tilted photos and 29 errors were related to photo resolution. These errors can be attributed to excessive rotation, scaling, zooming, and color adjustments during the segmentation process. Therefore, it is deemed that implementing a system to detect and request the re-uploading of excessively tilted or low-resolution photos when athletes utilize the system could resolve such issues.

The significance of this study lies in the development of a doping drug-recognition system that can relatively easily classify banned substances, which some athletes may face difficulties in identifying and classifying, through deep learning-based technology. Further research is expected to improve system performance and allow it to more clearly detect banned substances. One suggestion is for researchers to continuously update the system with the annual list of banned substances and the built system database. Finally, the doping drug-recognition system, if successfully commercialized, can serve as an effective option for the prevention of the use of banned substances among athletes.

## Figures and Tables

**Table 1:** Data search sources.

Data Search	Source
Google	https://www.google.co.kr/ (accessed on 20 March 2023)
Korea Pharmaceutical Information Center	https://www.health.kr/main.as (accessed on 20 March 2023)
WADA	https://www.wada-ama.org/en (accessed on 20 March 2023)

**Table 2:** Preliminary test for text recognition accuracy by Google Tesseract OCR.

	Number of Drug Substances	Correct	Error	Accuracy
Google Tesseract OCR	323	311	12	96.3%

**Table 3:** Example of accuracy calculations based on a binary classification table.

Binary Classification		Reference Classification	
		Banned	Safe or Acceptable
Prediction categories	Banned drugs	True master (TM) 10	False master (FM) 2
	Acceptable drugs	False non-master (FN) 0	True non-master (TN) 10
Calculation for the system’s classification accuracy			
1. Accuracy: Accuracy refers to the frequency at which banned drugs are correctly recognized (true master [TM]) and acceptable drugs are correctly recognized (true non-master [TN]) in all prescription and drug substance images.			
An example of the formula used to calculate accuracy derived from this study is as follows. (TM + TN)/(TM + FN + FM + TN) = (10 + 10)/(10 + 0 + 2 + 10) = 0.9			
2. Sensitivity: Sensitivity refers to the percentage of banned substances’ images that are correctly classified as banned (TM).			
An example of the formula used to calculate sensitivity derived from this study is as follows. TM/(TM + FN) = 10/(10 + 0) = 1.0			
3. Specificity: Specificity refers to the percentage of acceptable substances’ images that are correctly classified as acceptable (TN).			
An example of the formula used to calculate specificity derived from this study is as follows. TN/(FM + TN) = 10/(2 + 10) = 0.83			

**Table 4:** Procedure of use of the developed doping drug-recognition system.

	**Step 1**	**Step 2**
Use procedure	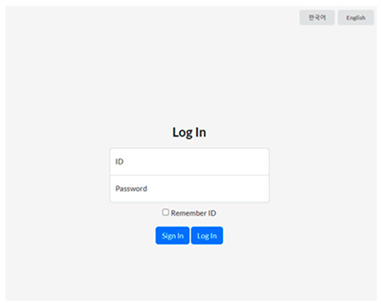	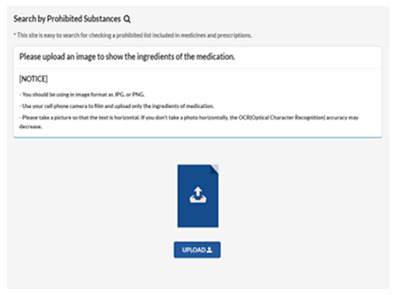
	Log in the system	Enter a drug substance image
	**Step 3**	**Step 4**
	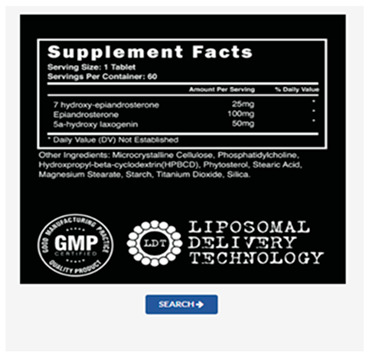	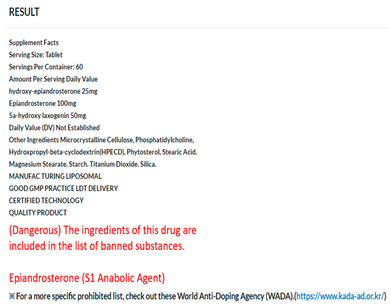
	Analyze substances after image upload	Produce the text as an output of analysis

**Table 5:** Character recognition accuracy of the developed doping drug-recognition system.

	Frequency of Words Extracted (*n*)	%
Total	5379	100
Error	91	1.6
Accuracy	98.3%	

**Table 6:** Validation of the system based on a binary classification table.

Binary Classification		Reference Classification	
		Banned	Safe or Acceptable
Prediction categories	Banned drugs	True master (TM) 218	False master (FM) 44
	Acceptable drugs	False non-master (FN) 0	True non-master (TN) 624
**Accuracy of the Doping Drug-Recognition System’s Classification**			
Accuracy			0.95
Sensitivity			1.00
Specificity			0.93

## Data Availability

Data is available by request from the corresponding author.
